# Huge toxic goiter extending to the posterior mediastinum; Case report with literature review

**DOI:** 10.1016/j.ijscr.2019.08.016

**Published:** 2019-08-20

**Authors:** Abdullah Saeed Abdullah, Alaa S. Bahjat, Ayad Ahmad Mohammed

**Affiliations:** a Duhok Kidney Transplantation Center, Duhok city, Kurdistan region, Iraq; b University of Duhok, College of Medicine, Department of Surgery, Duhok city, Kurdistan region, Iraq

**Keywords:** Goiter, Retrosternal goiter, Thyroidectomy, Manubriotomy, Sternotomy

## Abstract

**Introduction:**

Retrosternal goiter may occur in up to 7% of the cases, most of them extend to the anterior mediastinum, extension to the posterior mediastinum is very rare. It causes compression on mediastinal structures such as the trachea, the bronchi, the esophagus, and great vessels. The diagnosis is done mostly by CT scan. Most cases need surgery which is done by the combined cervical and the thoracic incisions.

**Case presentation:**

A 70-year-old man had history of thyroid enlargement for 10 years which was hyper-functioning and controlled with medical therapy. For the last 2 months the patient was complaining from dyspnea especially during supine posture and dysphagia. CT-scan showed huge extension of the thyroid gland to the posterior mediastinum causing compression over the tracheal and the esophagus. Surgery done through both cervical incision and manubriotomy and the huge thyroid gland extracted. The patient had uneventful recovery with no postoperative complications.

**Conclusion:**

Retrosternal goiter causes airway compromise in most patients. Surgery for such cases is a challenging procedure with higher rate of complications, CT-scan is the most single valuable tool in selecting patients for sternotomy before surgery but the final decision for sternotomy is best done during surgery. A team work between a thyroid surgeon and a thoracic surgeon has better results.

## Highlights

• Retrosternal goiter is defined when the thyroid gland extends below the thoracic inlet.
• It causes compression over the respiratory and the digestive passages, or pressure over the superior vena cava.
• It needs surgical treatment in most patients and usually requires the combined cervical and the thoracic approach.

## 1 Introduction

Retrosternal goiter is defined as any thyroid extension below the thoracic inlet or defined by some authors when more than 50% of its volume is present below this level [1].

The incidence goiter in general has decreased due to the use of the ionized salt but it may be seen in some parts of the world especially the mountain areas. The first description of the retrosternal extension of the thyroid gland was done in 1749, retrosternal goiter has been reported to occur in up to 7% of the cases. The majority of them extend to the anterior mediastinum, extension to the posterior mediastinum is very rare and occur in around 10% of all mediastinal extensions [1,2].

Extension of the enlarged thyroid to the posterior mediastinum cause a variety of symptoms which are mostly due to compression on the various mediastinal structures such as the trachea and the bronchi resulting in stridor and dyspnea, the esophagus causing dysphagia, and the great vessels resulting in facial congestion and palpitation [3].

The diagnosis of this condition is done by radiological evaluation mostly by CT scan, needle aspiration for histopathological evaluation is better to be avoided because of its dangerous location, it may induce hemorrhage inside the thyroid gland causing sudden increase in its size and acute respiratory distress [2].

Most cases need surgery which is done by the combined cervical and the thoracic incisions, during surgery the anesthetist should be aware because of the airway compression and the difficult intubation. The surgical outcome is excellent in most patients and long term follow up is not indicated [2].

The work of this case report has been reported in line with the SCARE criteria [4].

### 1.1 Patient information

A 70-year-old man had history of thyroid enlargement for 10 years presented to the surgical private clinic complaining from palpitation and weight loss.

### 1.2 Clinical findings

During examination the pulse rate 90 beats per minute with regular rhythm, and the blood pressure was 150/90 mmHg.

Neck examination showed big goiter extending to the retrosternal region with no eye signs. The reflexes were hyperactive and the patient experience weight loss which was not recorded.

### 1.3 Diagnostic assessment

The investigations showed low THS and elevated free T4 levels which was consistent with hyperthyroidism. The patient received antithyroid medications for 2 years and the over function of the thyroid gland was controlled.

During the last 2 months the patient was complaining from dyspnea especially during supine posture and dysphagia.

**Fig. 1:**
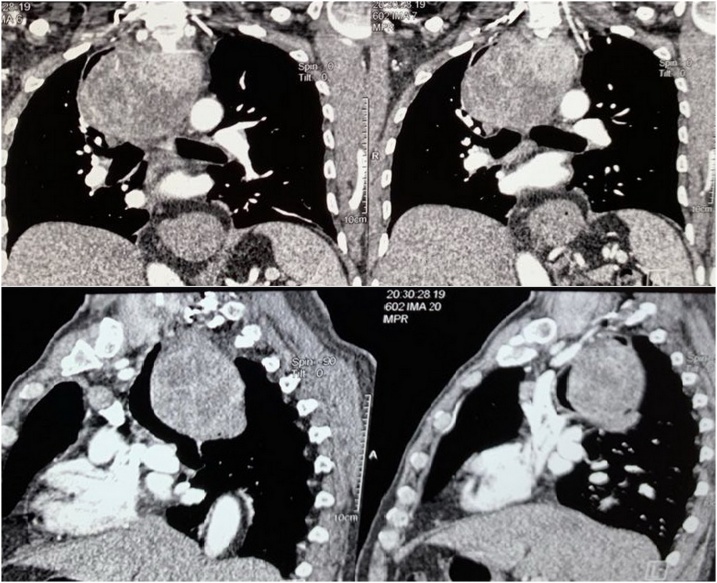
CT scan of the chest showing the enlarged thyroid gland extending to the posterior mediastinum and cussing pressure and shifting of the trachea and the esophagus.

CT-scan of the neck and the chest showed huge extension of the thyroid gland to the posterior mediastinal space and causing deviation of compression of both the tracheal and the esophagus Fig. 1.

**Fig. 2:**
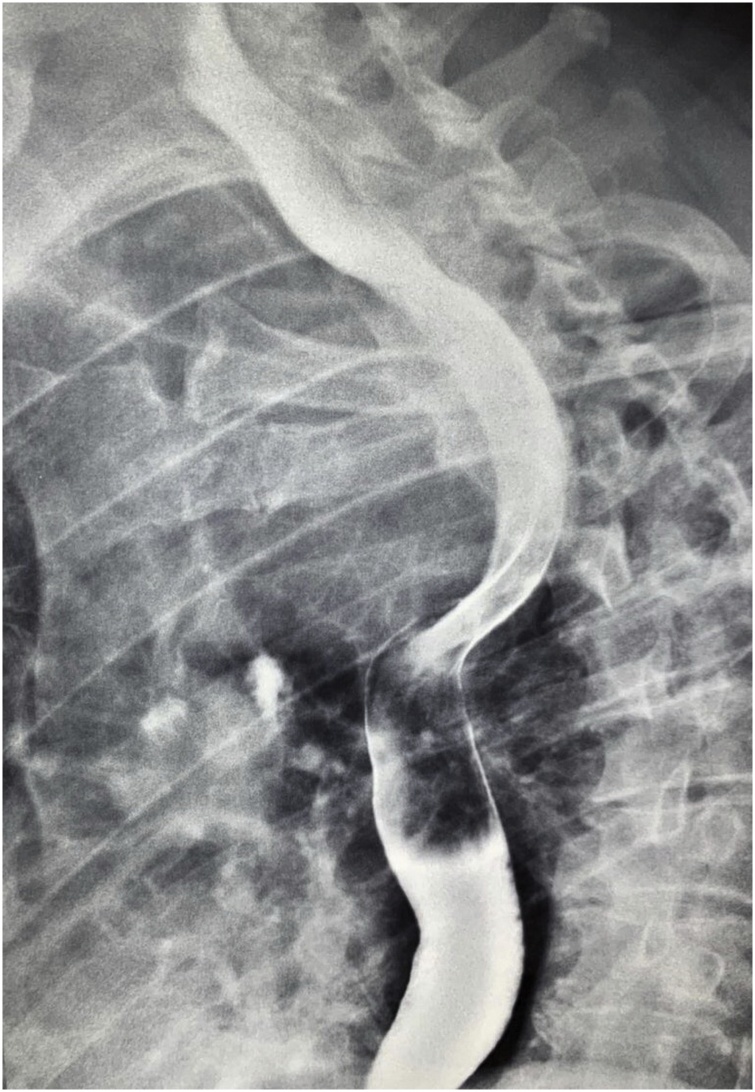
Barium study showing the deviation of the esophagus by the large thyroid gland.

The esophageal deviation was evident also during performing barium study of the upper gastrointestinal tract Fig. 2.

### 1.4 Therapeutic intervention

**Fig. 3:**
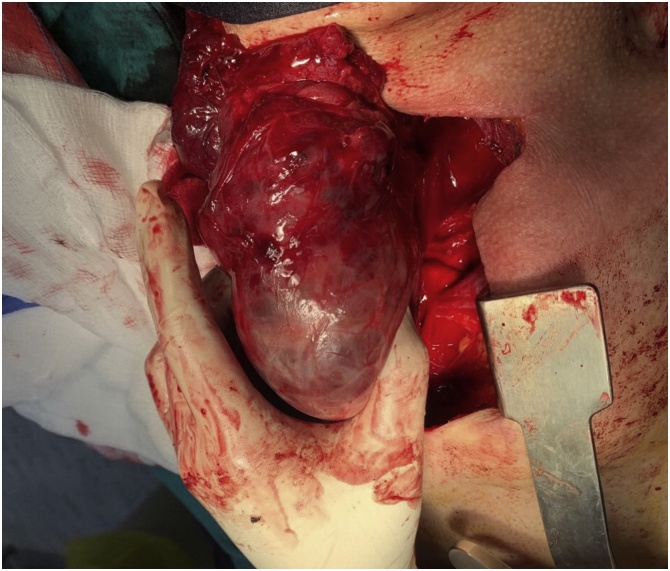
Intraoperative picture showing the large thyroid gland extracted from the upper mediastinum after performing sternotomy.

**Fig. 4:**
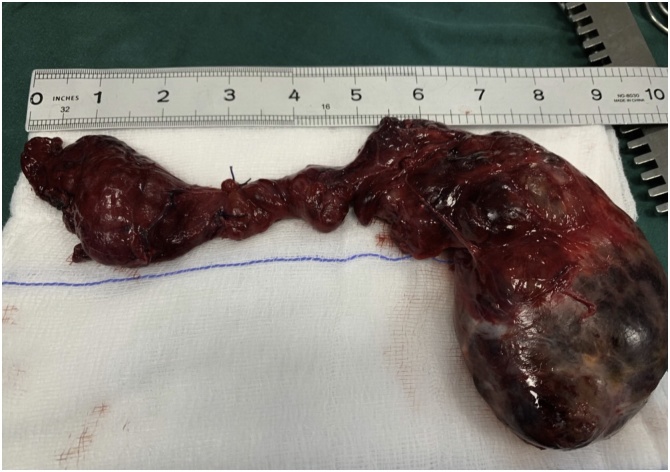
showing the size of the thyroid gland (in inches) after being completely removed.

Surgery was done through collar incision (cervical incision) and sternotomy (manubriotomy) and the huge thyroid gland extracted and removed completely and sent for histopathological study which showed a benign thyroid enlargement with colloid degeneration Fig. 3, Fig. 4.

### 1.5 Follow-up and outcomes

The patient had uneventful recovery with no voice changes and the 4 -h postoperative serum parathyroid hormone level and serum calcium were with normal values. The patient discharged after 3 days and 2 low pressure suction drains remained in place for 5 days. Follow up done for 2 months with no reported complications and the patient received thyroid replacement therapy.

## 2 Discussion

The posterior mediastinal goiter is very rare and sometimes cases of ectopic mediastinal goiter have been reported in extremely rare occasions [2,5].

Patients may be completely asymptomatic but most patients have compression symptoms. The level of the compression is usually at the thoracic inlet but some cases have lower levels of compression. Respiratory symptoms like stridor, dyspnea and wheezes are the most common symptoms, dysphagia may occur in cases of esophageal compression which may be seen in up to 25% of the patients, rarely there may be compression of the superior vena cava resulting in facial congestion during elevation of the upper limbs [6].

Some cases may be treated wrongly as bronchial asthma before being diagnosed with retrosternal goiter and in some rare cases respiratory failure have been reported. Sudden respiratory deterioration may be caused by hemorrhage inside one of the cysts [7].

CT scan of the chest is very helpful in patients with goiter extending to the chest because it confirms the extension and its level by clarifying the continuity with the cervical one, shows the architecture and the borders of the thyroid, and the presence of calcifications [8].

Surgery is challenging in such cases and the majority of patents needs the combined approach for surgery, i.e. the cervical and the thoracic approach by sternotomy which may be manubriotomy or total sternotomy, some cases may need thoracotomy which provides better visualization over sternotomy, there is no clear criteria defining which patient will need sternotomy or thoracotomy but cases with very large gland, extension to the posterior mediastinum, recurrent thyroid with previous transcervical approach, or suspicion of malignancy in retrosternal goiter mostly need the combined procedure [[1], [2], [3],^7^].

The differential diagnoses of posterior mediastinal goiter include congenital foregut cysts which contain calcium, esophageal tumors, neurogenic tumors, bronchogenic cysts or carcinoma, lymphomas, large calcified lymph node, vertebral tumors, and hamartomas [6].

Complications of surgery may include injury to the adjacent anatomical structures such as the recurrent laryngeal nerves which should be visualized during surgery, bleeding and hematoma, hypocalcemia, tracheal injury, tracheomalacia, pneumothorax, esophageal injury, phrenic nerve injury, and injury to the intrathoracic vessels [2,6,7].

Our case represented one of the very rare presentation of retrosternal goiter which was treated successfully with the combined cervical and the thoracic approach.

## Sources of funding

None.

## Ethical approval

Ethical approval has been exempted by my institution for reporting this case.

## Consent

Written informed consent was obtained from the patient for publication of this case report and accompanying images.

## Author contribution

Dr Abdullah Saeed Abdullah and Dr Alaa S. Bahjat are the surgeons who performed the operation.

Dr Abdullah Saeed Abdullah and Dr Ayad Ahmad Mohammed contributed to the concept of reporting the case and the patient data recording.

Drafting the work, design, and revision done by Dr Ayad Ahmad Mohammed.

Dr Abdullah Saeed Abdullah took the consent from the patient for publishing the case.

Final approval of the work to be published was done by Dr Abdullah Saeed Abdullah and Dr Ayad Ahmad Mohammed.

## Registration of research studies

This work is case report and there is no need of registration

## Guarantor-

Dr Ayad Ahmad Mohammed is guarantor for the work

## Provenance and peer review

Not commissioned, externally peer-reviewed

## Declaration of Competing Interest

The author has no conflicts of interest to declare
